# LH level on ovulation trigger day has a different impact on the outcomes of agonist and antagonist regimens during in vitro fertilization

**DOI:** 10.1186/s13048-023-01110-8

**Published:** 2023-01-27

**Authors:** Xi Luo, Bo Deng, Lei Li, Rui Ma, Xuancheng Mai, Ze Wu

**Affiliations:** 1 Department of Reproductive Medicine, The First People’s Hospital of Yunnan Province, Kunming, China; 2 Reproductive Medical Center of Yunnan Province, The Affiliated Hospital of Kunming University of Science and Technology, Kunming, China; 3 NHC Key Laboratory of Periconception Health Birth in Western China, Kunming, China; 4 Faculty of Life Science and Technology, Kunming University of Science and Technology, Kunming, China; 5 Medical School, Kunming University of Science and Technology, Kunming, China

**Keywords:** Luteinizing hormone, Assisted reproductive technology, Clinical outcome, Clinical pregnancy, Live birth

## Abstract

**Background:**

To assess the impact of the luteinizing hormone level on ovulation trigger day (LHOTD) on in vitro fertilization (IVF) outcomes in gonadotropin-releasing hormone (GnRH) agonist and antagonist regimens during fresh embryo transfer cycles.

**Methods:**

A stepwise, progressive multivariate regression model was introduced to assess the effect of the LHOTD on clinical pregnancy and live birth rates. Mantel‒Haenszel stratification analysis was used to examine the association between the LHOTD and clinical outcomes with the antagonist regimen.

**Results:**

The LHOTD had different distributions in the agonist and antagonist regimens. The cycles were assigned into three LHOTD tertile groups. In the agonist regimen, compared with the 1^st^ tertile (T1), in the 2^nd^ (T2) and 3^rd^ (T3) tertiles, the adjusted odds ratios (ORs) (95% confidence intervals [CIs], *P value*) were 1.187 (1.047–1.345, 0.007) and 1.420 (1.252–1.610, < 0.001) for clinical pregnancy, respectively, and 1.149 (1.009–1.309, 0.036) and 1.476 (1.296–1.681, < 0.001) for live birth. In the antagonist regimen, there was no significant difference in clinical pregnancy and live birth rates among the tertiles. However, in the stratified group of patients aged less than 35 years, the ORs (95% CIs, *P value*) of T2 and T3 were 1.316 (1.051–1.648, 0.017) and 1.354 (1.077–1.703, 0.009) for clinical pregnancy, respectively, and 1.275 (1.008–1.611, 0.043) and1.269 (0.999–1.611, 0.051) for live birth. Moreover, there was a discrepancy in the results among the subdivided LHOTD T1 groups adopting the antagonist regimen. Compared with that of the < 1.06 mIU/mL subgroup, the ORs (95% CIs, *P value*) of the > 1.5 mIU/mL subgroup were 1.693 (1.194–2.400, 0.003) for clinical pregnancy and 1.532 (1.057–2.220, 0.024) for live birth after eliminating potential confounders.

**Conclusions:**

The LHOTD was profoundly suppressed in the agonist regimen, and its level was positively correlated with clinical pregnancy and live birth rates. In contrast, in the flexible antagonist regimen, the LHOTD was significantly higher than that in the agonist regimen and did not correlate with the outcome, except for women in the nonadvanced age group and those with an excessively suppressed LHOTD. Further investigation is required to determine the rationale for these findings.

## Introduction

Gonadotropins include two pivotal reproductive hormones: follicle-stimulating hormone (FSH) and luteinizing hormone (LH). During in vitro fertilization and embryo transfer (IVF-ET) therapy, FSH is used for ovarian stimulation, but the role of LH, as well as the timepoint and criteria to add LH in the controlled ovarian stimulation (COS) process, remains controversial [1–3]. Although LH is known to promote follicular maturation and induce ovulation [4], its other functions in the reproductive process remain unclear.

After combining with the luteinizing hormone/chorion gonadotropin receptor (LHCGR) in the cell membrane, LH can activate the downstream signaling pathway [5]. The LHCGR is primarily expressed in follicular granulosa cells and can promote hormone-dependent steroidogenesis [6]. However, since the LHCGR is also found in nongonadal but reproductively relevant tissue, such as uterine tissues, LH might be involved in roles other than follicular growth [7]. Therefore, additional clinical and biological studies are required to explore the functions of LH to better understand the entire reproductive process.

During IVF-ET therapy, FSH is used to stimulate follicular growth. With follicular development, the serum LH level can increase and may cause a premature LH surge and ovulation, which can be suppressed by gonadotropin-releasing hormone (GnRH) analogs (commonly agonists and antagonists) [8]. The mechanisms of IVF protocols using GnRH analogs as agonists or antagonists are different. When a GnRH analog is administered as an agonist, there is a flare-up of FSH and LH, but the GnRH analog receptors are later downregulated to a low level in the pituitary gland [9]. Endogenous gonadotropins remain at a lower level and for a longer duration with agonist use compared to antagonist use. When GnRH is used as an antagonist, it can rapidly inhibit gonadotropin secretion by reverse binding to the GnRH receptor [10]. Thus, its advantage is rapid and temporary suppression of pituitary secretion. Therefore, the LH level may vary and play different roles depending on the IVF regimen used.

To explore the effect of different regimens on the LH level and clinical outcome, we herein compared the LH distribution of agonist and flexible antagonist regimens and assessed the effects of the LH level on the ovulation trigger day (LHOTD) on the overall clinical outcome of both regimens.

## Materials and methods

### Study design and participants

This retrospective cohort study was conducted after approval by the ethics committee of the First People’s Hospital of Yunnan Province (No. KHLL2020-KY013). Patients undergoing their first cycle with controlled ovarian stimulation using a GnRH analog and fresh embryo transfer (ET) at the university-affiliated hospital from January 2017 to May 2020 were included. The exclusion criteria were as follows: 1) the presence of polycystic ovary syndrome (PCOS), luteinized unruptured follicle syndrome, and other endocrinology disorders; 2) the use of oral contraceptives in the last 3 months; 3) a self-reported history of a family genetic disorder or an abnormal chromosomal karyotype; and 4) incomplete medical records. The clinical information was deidentified. Simple randomization was used to select the agonist or antagonist regimen. However, for patients with normal ovarian reserve and the desire to have more oocytes retrieved, the agonist tended to be used. For patients with a potential high ovarian response or low anti-Müllerian hormone (AMH) level (less than 2 ng/mL), antagonists tended to be used. Finally, pituitary suppression was initiated after the patient learned about the difference between agonists and antagonists and signed the informed consent form.

### Agonist regimen procedure

The agonist pituitary suppression regimen was initiated at the mid-luteal phase. A total of 1.25 mg of the long-acting triptorelin acetate (Diphereline, Ipsen Pharma Biotech, Signes, France; Decapeptyl, Ferring GmbH, Kiel, Germany) was injected intramuscularly (IM). After approximately 14 days, when the estradiol (E2) level was < 50 pg/mL, the endometrial thickness was < 5 mm, and no ovarian cysts were noted, COS was initiated by an FSH (rFSH, Gonal-F, Merck-Serono, Aubonne, Switzerland) subcutaneous (SC) injection once a day. Ultrasound and serum hormone examinations were conducted from the 6^th^ day of COS. When there were two dominant follicles with a diameter of > 18 mm, the serum LH, E2, and progesterone levels and the endometrial thickness (EMT) were recorded. Then, 5,000 IU of exogenous human chorionic gonadotropin (hCG) was injected at 10 PM to trigger the ovulation process.

### Antagonist regimen procedure

In the flexible antagonist regimen, exogenous FSH (rFSH, Gonal-F, Merck-Serono, Aubonne, Switzerland) was SC injected daily from the 2^nd^ day of menses to start COS. From the 4^th^ day, when the E2 level was > 300 pg/mL, the diameter of the dominant follicle was > 14 mm or the LH level was > 10 mIU/mL, a 0.25 mg SC injection of cetrorelix acetate (Cetrotide, Merck Europe B.V., Idron, France) or ganirelix acetate (Orgalutran, Merck Sharp & Dohme B. V., Ravensburg, Germany) was injected daily until the diameters of the two dominant follicles were > 18 mm. Subsequently, the ovulation trigger was administered by injecting exogenous hCG.

### IVF/ICSI-ET

Thirty-six hours after the ovulation trigger was administered, transvaginal ultrasound-guided puncture and oocyte retrieval were performed. According to male sperm motile measurements, conventional IVF or ICSI was conducted. The statuses of the zygote and embryo were monitored and recorded. The presence of two pronuclei (2PN) on the first day was considered normal fertilization. The number of 2PN divided by the number of oocytes retrieved was defined as the 2PN rate. On the 3^rd^ day, the original 2PN embryos, with 6–8 blastomeres and cell debris < 20%, were considered the optimal choice for ET. β-hCG was tested on the 14th day after ET. Luteal support was continued to the 8^th^ week if the β-hCG test was positive.

### Data collection

Baseline characteristics, including age, body mass index (BMI), AMH level, and antral follicle count (AFC), were recorded. Primary or secondary infertility, causes of female infertility, history of parturition or miscarriage, and methods of fertilization (conventional IVF versus ICSI) were recorded. The LHOTD was collected on the day of the ovulation trigger, and all serum hormones were measured by a UniCel DxI 800 Access Immunoassay System (Beckman Coulter, CA, USA). Medical records were also reviewed to document the levels of estradiol and progesterone, as well as the endometrial thickness, on the ovulation trigger day.

After ET, follow-up visits were cancelled if the β-hCG test was negative. If the β-hCG test was positive, a clinical pregnancy test was conducted at the 5^th^–6^th^ week. The detection of a viable sac(s) was defined as a clinical pregnancy. An extrauterine sac was defined as an ectopic pregnancy. Pregnancy loss during the first trimester was defined as early pregnancy loss. If pregnancy continued past the 12^th^ week, it was defined as an ongoing pregnancy. The primary outcomes of the study were the clinical pregnancy and live birth rates.

### Statistical analysis

Statistical analyses were conducted with SPSS (version 26.0, Armonk, NY, USA). A *P value* of < 0.05 via a two-tailed test was considered statistically significant. The cycles were assigned into tertile groups based on the LHOTD in each agonist and antagonist regimen (T1, T2, and T3). To compare the three LHOTD groups, normally distributed continuous data, such as age, BMI and the number of oocytes retrieved, are expressed as the mean ± standard deviation (SD) and were compared by one-way ANOVA. Nonnormally distributed continuous data, such as the AMH level, AFC, indicators on ovulation trigger day and 2PN rate, are expressed as the median (25^th^–75^th^ percentiles) and were compared by the Kruskal–Wallis test. Categorical data, such as gravidity and parity, medical history, fertilization method, the number of embryos transferred and clinical outcomes, are demonstrated as counts (percentage) and were analyzed by the chi-square (χ^2^) test. The multiple pairwise comparison *P value* was adjusted by the Bonferroni method. A stepwise progressive multivariate regression model [11] was introduced to assess the effect of the LHOTD on the clinical pregnancy and live birth rates. Finally, a total of 3 models were developed to account for the important information of each IVF-ET treatment stage as comprehensively as possible to eliminate confounding factors. Model 1 included age, BMI, the AMH level, and the AFC. In Model 2, primary infertility, the cause of female infertility, and history of parturition or miscarriage were included. In Model 3, we added the ovulation trigger day indicators, including progesterone, E2, EMT, the number of oocytes retrieved, fertilization method, and the number of embryos transferred, on the basis of Model 2. Mantel‒Haenszel stratification analysis [12] was used to demonstrate that the LHOTD of the antagonist regimen was not correlated with the clinical outcome.

## Results

**Fig. 1:**
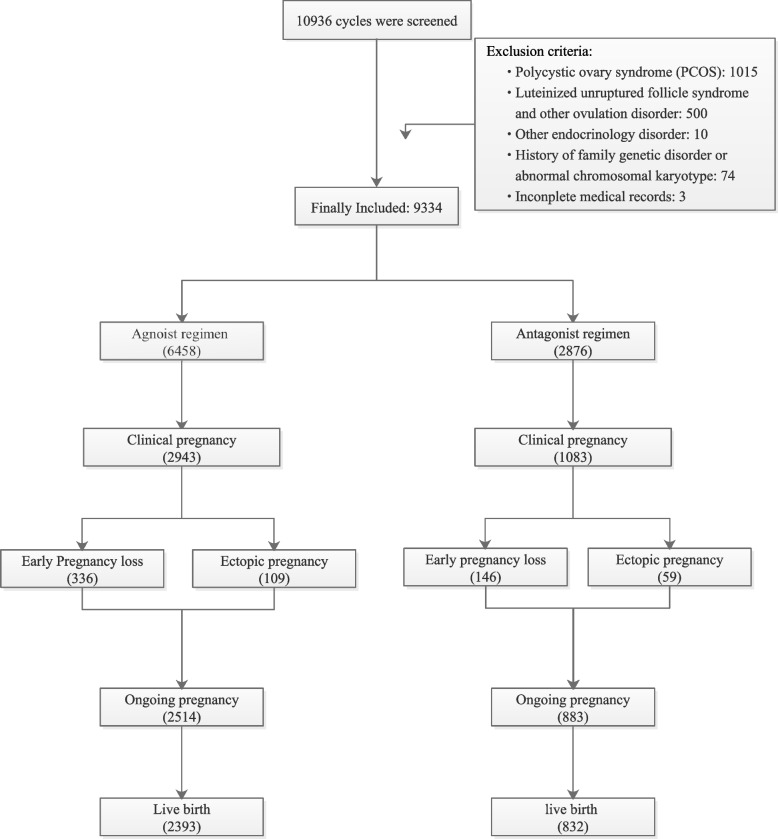
The flow chart of patients’ enrollment

**Fig. 2:**
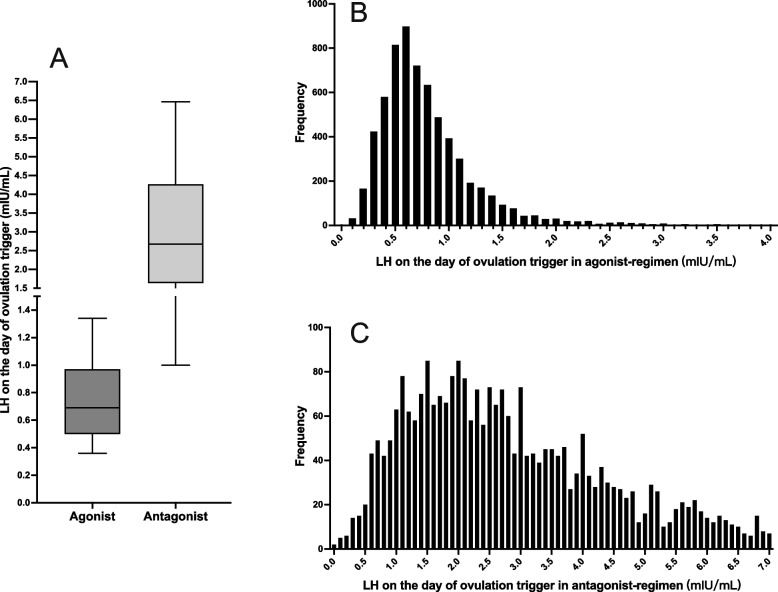
Distribution of LHOTD in agonist and antagonist regimens. **A** Box-whisker chart of LHOTD in agonist and antagonist regimens. The lower whisker is the 10^th^ percentile of LHOTD, and the upper whisker is the 90^th^ percentile of LHOTD. The lower and upper lines of the box plot indicate the 25^th^ and 75^th^ percentiles, respectively, and the middle line is the median. **B** and **C** Histograms of LHOTD in agonist and antagonist regimens. LH: Luteinizing hormone, LHOTD: Luteinizing hormone on the ovulation day

**Fig. 3:**
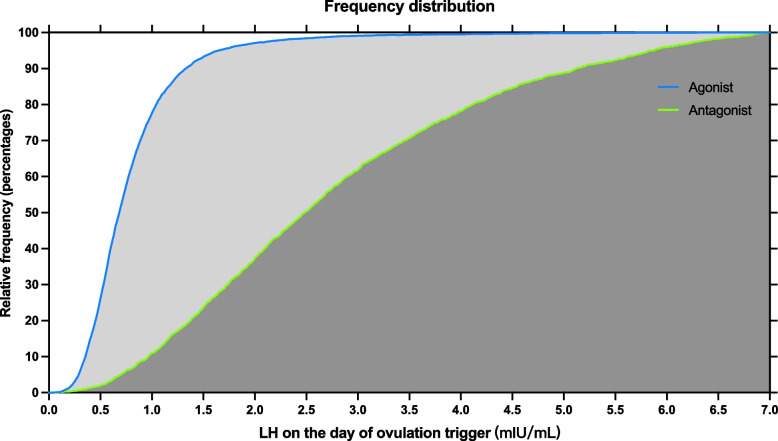
Cumulative frequency distribution of LHOTD in agonist and antagonist regimens. The blue and green lines represent the relative cumulative frequency percentages, respectively. LHOTD: Luteinizing hormone on the ovulation day, LH: Luteinizing hormone

A total of 9,334 fresh COS and ET cycles were identified, including 6,458 with agonist regimens and 2,876 with antagonist regimens (Fig. 1). The LHOTD distributions were significantly different between the agonist and antagonist regimens (Figs. 2 and 3). The medians (25^th^–75^th^ percentiles) of the agonist and antagonist regimens were 0.69 (0.5–0.97) mIU/mL and 2.67 (1.64–4.27) mIU/mL, respectively. The LHOTD of the agonist regimen had a more concentrated distribution and a lower level than that of the antagonist regimen. Ninety percent of patients in the agonist regimen group had an LHOTD of < 1.34 mIU/mL, whereas 90% of patients in the antagonist regimen group had an LHOTD > 1 mIU/mL. These results implied that the agonist regimen might more profoundly suppress pituitary secretion.

### Demographic analysis of the LHOTD tertile groups in the agonist and antagonist regimens

Table 1 summarizes the demographic characteristics of the LHOTD tertile groups, and we noted that the characteristic trends in the LHOTD tertile groups differed between the agonist and antagonist regimens. With agonist use, the LHOTD was negatively correlated with age, BMI, and vaginal delivery history but positively correlated with the AFC and E2 levels. However, with antagonist use, the LHOTD with antagonist showed an opposite trend relative to the aforementioned variables except for vaginal delivery history and E2 level, while AMH and progesterone levels showed similar trends between the agonist and antagonist regimens (i.e., the LHOTD correlated negatively with the AMH level and positively with the progesterone level).

**Table 1:** Demographic analysis of the LHOTD tertile groups with agonist and antagonist regimens

Variables	Agonist regimen				Antagonist regimen			
	T1 (< 0.56) *N* = 2113	T2 (0.56–0.84) *N* = 2159	T3 (> 0.84) *N* = 2186	*P*-value	T1 (< 1.98) *N* = 956	T2 (1.98–3.61) *N* = 959	T3 (> 3.61) *N* = 961	*P*-value
Age, years	31.20 ± 4.19 ^a^	30.91 ± 4.05 ^a^	30.75 ± 4.31 ^b^	0.002	31.83 ± 5.10 ^a^	32.90 ± 5.21 ^b^	33.62 ± 5.01 ^c^	< 0.001
BMI	22.32 ± 2.72	22.04 ± 2.70	21.80 ± 2.63	< 0.001	22.10 ± 2.82 ^a^	22.36 ± 2.71 ^a, b^	22.50 ± 2.73 ^b^	0.006
AMH, ng/mL	3.14 (2.06–4.69) ^a^	3.12 (2.08–4.56) ^a, b^	3.04 (1.98–4.40) ^b^	0.037	2.64 (1.47–5.10) ^a^	2.31 (1.25–4.20) ^b^	1.88 (1.02–4.08) ^c^	< 0.001
AFC	10 (8–12) ^a^	11 (8–12) ^a, b^	11 (9–13) ^b^	< 0.001	10 (7–14) ^a^	9 (6–12) ^b^	8 (6–12) ^c^	< 0.001
Primary infertility, n (% of total)	878 (41.6) ^a^	976 (45.2) ^b^	970 (44.4) ^a, b^	0.042	411 (43.0) ^a^	351 (36.6) ^b^	345 (35.9) ^b^	0.002
Cause of female infertility, n (% of total)								
Normal (Male factor only)	273 (12.9)	297 (13.8)	314 (14.4)	0.050	131 (13.7) ^a^	94 (9.8) ^b^	95 (9.9) ^b^	0.315
Fallopian tube factors	1689 (79.9)	1667 (77.2)	1682 (76.9)		747 (78.1) ^a^	793 (82.7) ^b^	783 (81.5) ^a, b^	
Endometriosis	22 (1.0)	27 (1.3)	29 (1.3)		9 (0.9)	10 (1.0)	9 (0.9)	
Other factors	5 (0.2)	7 (0.3)	7 (0.3)		5 (0.5)	3 (0.3)	6 (0.6)	
Unknown reason	86 (4.1)	88 (4.1)	78 (3.6)		40 (4.2)	37 (3.9)	42 (4.4)	
Multi factors	38 (1.8) ^a^	73 (3.4) ^b^	76 (3.5) ^b^		24 (2.5)	22 (2.3)	26 (2.7)	
History of parturition or miscarriage, n (% of total)								
miscarriage	475 (22.5)	484 (22.4)	488 (22.3)	0.992	218 (22.8)	232 (24.2)	244 (25.4)	0.416
cesarean section	68 (3.2)	60 (2.8)	69 (3.2)	0.663	22 (2.3) ^a^	38 (4.0) ^a, b^	43 (4.5) ^b^	0.028
vaginal delivery	162 (7.7) ^a^	137 (6.3) ^a, b^	109 (5.0) ^b^	0.001	67 (7.0)	89 (9.3)	93 (9.7)	0.081
Method of fertilization, n (% of total)								
Conventional IVF	1741 (82.4)	1783 (82.6)	1756 (80.3)	0.103	763 (79.8)	802 (83.6)	795 (82.7)	0.075
ICSI	372 (17.6)	376 (17.4)	430 (19.7)		193 (20.2)	157 (16.4)	166 (17.3)	
Indicators on the day of ovulation trigger								
Luteinizing hormone, mIU/mL	0.42 (0.33–0.49) ^a^	0.68 (0.61–0.76) ^b^	1.12 (0.96–1.41) ^c^	< 0.001	1.30 (0.93–1.64) ^a^	2.67 (2.30–3.06) ^b^	5.22 (4.26–7.01) ^c^	< 0.001
Estradiol, pg/mL	2483 (1578–3609) ^a^	2529 (1689–3642) ^a, b^	2608 (1749–3657) ^b^	0.005	2070 (1182–3207)	1974 (1188–3130)	1917 (1222–3021)	0.437
Progesterone, ng/mL	0.92 (0.64–1.31) ^a^	0.97 (0.68–1.33) ^a, b^	0.99 (0.7–1.35) ^b^	< 0.001	0.90 (0.61–1.31) ^a^	0.93 (0.63–1.29) ^a^	1.03 (0.76–1.38) ^b^	< 0.001
Endometrial thickness, mm	11 (9–12) ^a^	11 (10–13) ^a, b^	11 (10–13) ^b^	0.010	10 (9–12)	10 (9–12)	10 (9–12)	0.886

T1, T2, and T3 are the 1^st^, 2^nd^, and 3^rd^ LHOTD tertile groups in two regimens, respectively

The superscript characters a, b, and c represent pairwise comparisons between two groups of the same character, with no significant difference

*BMI* Body mass index, *AMH* Anti-Müllerian hormone, *AFC* Antral follicular count, *IVF* In vitro fertilization, *ICSI* Intracytoplasmic sperm injection

### Clinical outcomes of the LHOTD tertile groups with agonist and antagonist regimens

There were some significant differences in the clinical outcomes of the three LHOTD groups in pairwise comparisons in both agonist and antagonist regimens (Table 2). In the agonist regimen, the major results showed that a higher LHOTD implied a smaller number of oocytes retrieved and higher clinical pregnancy and live birth rates. Furthermore, based on clinical-pregnancy-positive cycles, the rates of ongoing pregnancies were lower in T1 and T2 compared to T3.

**Table 2:** Clinical outcomes of the LHOTD tertile groups with agonist and antagonist regimens

Variables	Agonist regimen				Antagonist regimen			
	T1 (< 0.56) *N* = 2113	T2 (0.56–0.84) *N* = 2159	T3 (> 0.84) *N* = 2186	*P*-value	T1 (< 1.98) *N* = 956	T2 (1.98–3.61) *N* = 959	T3 (> 3.61) *N* = 961	*P*-value
Number of oocytes retrieved	11.04 ± 4.45 ^a^	10.88 ± 4.34 ^a^	10.57 ± 4.34 ^b^	0.001	9.00 ± 4.74 ^a^	8.22 ± 4.51 ^b^	7.23 ± 4.35 ^c^	< 0.001
2PN rate	57.1 (41.2–71.4)	55.6 (40–71.4)	57.1 (40–71.4)	0.396	52.9 (38.5–70.0)	55.6 (40.0–75.0)	55.6 (38.5–75.0)	0.062
Number of embryos transferred, n (% of total)								
1	258 (12.2)	255 (11.8)	276 (12.6)	0.714	175 (18.3) ^a^	186 (19.4) ^a^	235 (24.5) ^b^	0.002
2	1855 (87.8)	1904 (88.2)	1910 (87.4)		781 (81.7) ^a^	773 (80.6) ^a^	726 (75.5) ^b^	
Clinical pregnancy, n (% of ET cycles)	875 (41.4)	985 (45.6)	1083 (49.5)	< 0.001	344 (36.0)	370 (38.6)	369 (38.4)	0.424
Ectopic pregnancy, n (% of clinical pregnancy)	38 (4.3)	43 (4.4)	28 (2.6)	0.050	21 (6.1)	17 (4.6)	21 (5.7)	0.653
Early pregnancy loss, n (% of clinical pregnancy)	112 (12.8)	115 (11.7)	109 (10.1)	0.159	45 (13.1)	48 (13.0)	53 (14.4)	0.829
Ongoing pregnancy, n (% of clinical pregnancy)	730 (83.4)	831 (84.4)	953 (88)	0.009	279 (81.1)	307 (83.0)	297 (80.5)	0.664
Live births, n (% of ET cycles)	697 (33.0)	783 (36.3)	913 (41.8)	< 0.001	265 (27.7)	286 (29.8)	281 (29.2)	0.578

T1, T2, and T3 are the 1^st^, 2^nd^, and 3^rd^ LHOTD tertile groups in two regimens, respectively

The superscript characters a, b, and c represent pairwise comparisons between two groups of the same character, with no significant difference

*2PN* 2 pronuclear, *ET* Embryo transfer

However, with the antagonist regimen, the major differences were in the outcomes of ovarian stimulation. The results showed that a higher LHOTD was associated with a smaller number of oocytes retrieved and a larger proportion of single-embryo transfer cycles. There were no differences in clinical outcomes with respect to the clinical pregnancy and live birth rates, which was different from the agonist regimen.

### Multivariate logistic regression of clinical pregnancy and live birth rates with the agonist regimen

In the agonist regimen, there were significant differences in the clinical outcomes in the cross-tabular analysis. Due to the long duration of IVF therapy, to eliminate the confounders at different phases, a stepwise, progressive multivariate logistic regression was conducted (Table 3). The results demonstrated that the LHOTD was an independent factor affecting the clinical pregnancy and live birth rates in the fresh ET cycle of the GnRH-agonist regimen. Compared with T1, in T2, the adjusted ORs ranged from 1.167–1.187 for clinical pregnancy and 1.129–1.149 for live birth. The adjusted ORs of T3 ranged from 1.353–1.420 for clinical pregnancy and 1.408–1.476 for live birth. Comparing T1 versus T3, the differences in the clinical pregnancy and live birth rates were extremely significant, with a *P value* < 0.001.

**Table 3:** Stepwise multivariate logistic regression of the clinical pregnancy and live birth rates with the agonist regimen

Groups	Univariate regression		Model 1		Model 2		Model 3	
	Crude OR	*P*-value	Adjusted OR (95% CI)	*P*-value	Adjusted OR (95% CI)	*P*-value	Adjusted OR (95% CI)	*P*-value
Clinical pregnancy (Reference T1 [< 0.56, *n* = 2113])								
T2 (0.56–0.84, *n* = 2159)	1.187 (1.052–1.340)	0.006	1.167 (1.033–1.318)	0.013	1.172 (1.037–1.324)	0.011	1.187 (1.047–1.345)	0.007
T3 (> 0.84, *n* = 2186)	1.389 (1.231–1.567)	< 0.001	1.353 (1.198–1.529)	< 0.001	1.366 (1.208–1.544)	< 0.001	1.420 (1.252–1.610)	< 0.001
Live birth (Reference T1 [< 0.56, *n* = 2113])								
T2 (0.56–0.84, *n* = 2159)	1.156 (1.019–1.312)	0.024	1.129 (0.994–1.283)	0.062	1.137 (1.001–1.292)	0.049	1.149 (1.009–1.309)	0.036
T3 (> 0.84, *n* = 2186)	1.457 (1.287–1.650)	< 0.001	1.408 (1.241–1.597)	< 0.001	1.423 (1.254–1.615)	< 0.001	1.476 (1.296–1.681)	< 0.001

Model 1: Age, BMI, AMH, and AFC

Model 2: Model 1, primary or secondary infertility, cause of female infertility (reference to normal), and history of parturition or miscarriage

Model 3: Model 2, indicators on the day of ovulation trigger (progesterone, estradiol, endometrial thickness), number of oocytes retrieved, fertilization method, 2PN rate, and number of embryos of transferred

T1, T2, and T3 are the 1^st^, 2^nd^ and 3^rd^ LHOTD tertile groups in the agonist regimen, respectively

*OR* Odds ratio, *CI* Confidence interval

### Stratified analysis of the impact of the LHOTD on the clinical outcomes and live birth rates

In the antagonist regimen, there was no significant difference in either the clinical pregnancy or live birth rates, regardless of the LHOTD, as revealed by cross-tabular analysis. To explore whether statistically significant differences in clinical outcomes existed in the different variable stratifications, a Mantel‒Haenszel stratification analysis was performed to further validate these results (Table 4). There were multiple potential factors that affected the clinical outcome of IVF therapy, such as age, BMI, the AMH level, the AFC, the number of oocytes retrieved, and the number of embryos transferred. These factors were stratified, and to validate the effects of the LHOTD on the clinical outcomes of clinical pregnancy and live births, we utilized univariate logistic regression. The results showed that there was no correlation between the LHOTD and clinical outcomes in any stratifications other than that of the group aged less than 35 years. Compared to T1, the ORs (95% CIs, *P value*) of T2 and T3 for clinical pregnancy were 1.316 (1.051–1.648, 0.017) and 1.354 (1.077–1.703, 0.009), respectively; for live birth, they were 1.275 (1.008–1.611, 0.043) and 1.269 (0.999–1.611, 0.051). Despite having a *P value* near the marginal level, these results still suggested that for young women aged < 35 years, a higher LHOTD may be associated with better IVF outcomes. Moreover, even though in some stratification groups, there were some differences with *P value*s of less than 0.05, the *P* for trend was greater than 0.05 among the tertile groups.

**Table 4:** Mantel‒Haenszel stratification analysis of the impact of the LHOTD on the clinical outcomes and live birth rates

Groups	N	Clinical pregnancy (Reference T1)				Live birth (Reference T1)			
		T2 Crude OR (95% CI)	*P*-value	T3 Crude OR (95% CI)	*P*-value	T2 Crude OR (95% CI)	*P*-value	T3 Crude OR (95% CI)	*P*-value
Age, years									
< 35	1795	1.316 (1.051–1.648)	0.017	1.354 (1.077–1.703)	0.009	1.275 (1.008–1.611)	0.043	1.269 (0.999–1.611)	0.051
35–39	745	1.001 (0.678–1.478)	0.998	0.972 (0.662–1.429)	0.886	1.047 (0.677–1.62)	0.836	1.112 (0.726–1.703)	0.627
> 39	336	0.857 (0.41–1.79)	0.682	1.071 (0.535–2.146)	0.846	0.939 (0.365–2.417)	0.897	0.989 (0.399–2.452)	0.981
BMI									
< 18.5	218	0.76 (0.393–1.471)	0.416	2.116 (1.088–4.113)	0.027	0.884 (0.438–1.785)	0.732	1.651 (0.831–3.279)	0.152
18.5–24.9	2122	1.08 (0.87–1.34)	0.487	1.037 (0.834–1.288)	0.744	1.016 (0.806–1.281)	0.890	1.007 (0.799–1.271)	0.951
> 24.9	536	1.515 (0.979–2.344)	0.062	1.195 (0.775–1.841)	0.420	1.714 (1.077–2.729)	0.023	1.235 (0.774–1.972)	0.376
AMH, ng/mL									
< 1.1	612	0.795 (0.503–1.257)	0.327	1.186 (0.78–1.804)	0.424	0.784 (0.478–1.287)	0.336	1.203 (0.769–1.882)	0.418
≥ 1.1	2264	1.219 (0.995–1.494)	0.057	1.108 (0.899–1.366)	0.334	1.204 (0.969–1.495)	0.094	1.053 (0.841–1.318)	0.652
AFC									
< 7	772	1.011 (0.656–1.558)	0.962	1.281 (0.854–1.92)	0.232	1.554 (0.933–2.588)	0.090	1.7 (1.045–2.768)	0.033
≥ 7	2104	1.211 (0.983–1.492)	0.072	1.179 (0.952–1.46)	0.132	1.100 (0.883–1.369)	0.396	1.088 (0.869–1.362)	0.463
Number of oocytes retrieved									
< 4	480	0.990 (0.576–1.702)	0.971	1.073 (0.648–1.776)	0.784	0.951 (0.526–1.72)	0.867	1.020 (0.589–1.767)	0.944
4–10	1580	1.171 (0.909–1.507)	0.222	1.157 (0.898–1.489)	0.259	1.168 (0.891–1.532)	0.262	1.166 (0.889–1.529)	0.266
> 10	816	1.197 (0.867–1.655)	0.275	1.378 (0.973–1.952)	0.071	1.177 (0.839–1.652)	0.346	1.206 (0.837–1.738)	0.314
Number of embryos transferred									
1	596	1.115 (0.655–1.898)	0.689	1.320 (0.804–2.166)	0.272	1.200 (0.677–2.128)	0.533	1.121 (0.647–1.943)	0.683
2	2280	1.138 (0.93–1.392)	0.211	1.159 (0.944–1.422)	0.158	1.110 (0.896–1.374)	0.339	1.141 (0.919–1.417)	0.232

*BMI* Body mass index, *LH* Luteinizing hormone, *AMH* Anti-Müllerian hormone, *AFC* Antral follicular count, *OR* Odds ratio, *CI* Confidence interval

### Multivariate logistic regression of the clinical pregnancy and live birth rates with the subdivided T1 LHOTD groups undergoing the antagonist regimen

To further investigate the impact of a profoundly suppressed LHOTD on the clinical outcome, the T1 LHOTD group was subdivided by tertile into t1, t2 and t3. A stepwise progressive multivariate logistic regression was conducted (Table 5). Regarding clinical pregnancy, the > 1.5 mIU/mL group had a better outcome than the < 1.06 mIU/mL group in both univariate and multivariate analyses. The ORs ranged from 1.584–1.720, and all *P* values were less than 0.05. However, in univariate logistic regression, there was no significant difference in the live birth rate among the 3 tertile groups. Nonetheless, after adjusting for the characteristics of the therapeutic process, the adjusted ORs ranged from 1.484–1.562, and the *P* values in the 3 models were all less than 0.05. These results suggest that excessive suppression of LH levels may have an adverse effect on clinical outcomes in patients who adopt COS antagonist regimens.

**Table 5:** Stepwise multivariate logistic regression of the clinical pregnancy and live birth rates with the subdivided T1 LHOTD groups with the antagonist regimen

Groups	Univariate regression		Model 1		Model 2		Model 3	
	Crude OR	*P*-value	Adjusted OR (95% CI)	*P*-value	Adjusted OR (95% CI)	*P*-value	Adjusted OR (95% CI)	*P*-value
Clinical pregnancy (Reference t1 (< 1.06, *n* = 314))								
t2 (1.06–1.50, *n* = 310)	1.112 (0.796–1.554)	0.533	1.175 (0.834–1.654)	0.356	1.145 (0.808–1.622)	0.446	1.204 (0.843–1.721)	0.307
t3 (> 1.50, *n* = 332)	1.584 (1.147–2.187)	0.005	1.720 (1.231–2.402)	0.001	1.616 (1.151–2.27)	0.006	1.693 (1.194–2.4)	0.003
Live birth (Reference t1 (< 1.06, *n* = 314))								
t2 (1.06–1.50, *n* = 310)	1.088 (0.760–1.559)	0.645	1.167 (0.807–1.689)	0.412	1.141 (0.784–1.662)	0.490	1.198 (0.818–1.754)	0.354
t3 (> 1.50, *n* = 332)	1.400 (0.991–1.976)	0.056	1.562 (1.09–2.237)	0.015	1.484 (1.031–2.136)	0.034	1.532 (1.057–2.22)	0.024

Model 1: Age, BMI, AMH, and AFC

Model 2: Model 1, primary or secondary infertility, cause of female infertility (reference to normal), and history of parturition or miscarriage

Model 3: Model 2, indicators on the day of ovulation trigger (progesterone, estradiol, endometrial thickness), number of oocytes retrieved, fertilization method, 2PN rate, and number of embryos of transferred

t1, t2, and t3 are the 1^st^, 2^nd^ and 3^rd^ sub-divided tertile of T1 LHOTD group in the antagonist regimen, respectively

*OR* Odds ratio, *CI* Confidence interval

## Discussion

In the present study, we compared the clinical outcomes among three different LHOTD groups with agonist and antagonist regimens. The results showed a disparate correlation between the LHOTD and clinical outcomes in both regimens. With effective pituitary suppression, the LHOTD maintains a low level and dense distribution and is an independent factor affecting the clinical pregnancy and live birth rates in women receiving an agonist regimen. In contrast, the LHOTD in the flexible antagonist regimen group showed a relatively high level and scattered distribution compared with the agonist regimen group and exhibited no correlation with the clinical outcome overall.

The limitations of our study included its single-center research and retrospective design. However, based on the large sample size and multiple statistical methods used, bias and confounders were eliminated as much as possible. Furthermore, the use of a stepwise multivariate logistic regression method demonstrated that there were significant differences in both clinical pregnancy and live birth rates in the agonist regimen after eliminating confounders at different phases. Moreover, agonist and antagonist regimens were both included in the same period to analyze the correlation between the LHOTD and clinical outcomes. These results revealed the effects of different pituitary-suppression methods on patients undergoing IVF. However, the cause of these results remains unclear, and we intend to continue exploring the LH mechanism affecting outcomes in the future.

At present, whether LH is connected to IVF outcomes remains controversial, irrespective of the regimen used. Agonists were the first GnRH analogs and were introduced to suppress a premature LH surge in the pituitary gland. Due to the persistence of agonist desensitization, the serum LH level can be suppressed throughout the entire COS process. Westergaard et al. reported that the LH level on the 8^th^ day of ovarian stimulation was positively correlated with the clinical outcomes, with a high LH level associated with a low early miscarriage rate and a high live birth rate [13]. Similar results were also reported in a retrospective study by Humaidan et al., which showed that the serum LH level on the 8^th^ day of COS had a significant impact on the ovarian response and clinical outcomes, suggesting that the LH level should not be too low during COS [14]. Our previous study demonstrated that profoundly suppressed LH levels on the day of COS initiation were correlated with a higher early miscarriage rate and adverse IVF outcomes [15]. However, Balasch et al. showed that the LH level on the 7^th^ day of stimulation was not correlated with the clinical outcomes, including clinical pregnancy, early pregnancy loss, and ongoing pregnancy [16]. Furthermore, in a study of 246 cycles, the LH level during COS was not connected to the ovarian response and clinical outcomes [17]. Moreover, in a study by Esposito et al., the mean LH level in periovulation was not correlated with clinical pregnancy or spontaneous abortion [18]. Research on the effect of the LHOTD on clinical outcomes using an agonist regimen has rarely been previously reported. To the best of our knowledge, the present study is the first to report a positive correlation of the LHOTD with clinical outcomes using an agonist regimen.

Since the discovery of GnRH-antagonist function in pituitary suppression [19], the antagonist regimen has been gradually introduced into COS. It has the advantages of fewer injections for patients, shorter stimulation days, avoidance of the adverse effects of agonists [20], and adequate prevention regarding premature LH surges [21]. Furthermore, the clinical outcomes of COS are similar between agonist and antagonist regimens [22, 23]. However, the effect of the LHOTD on clinical outcomes using antagonist regimens remain controversial. The influences of LH have been reported at different stages of COS. On the COS initiation day, higher LH levels may be beneficial to endometrial maturation [24]. In contrast, other research reported a reduced chance of successful pregnancy when a higher LH level occurred during the early follicular phase [25]. In patients with PCOS, the Day 2 or 3 basal LH level was unrelated to the clinical outcome [26]. At the mid-follicular phase, a study reported that the profound suppression of LH leads to a higher ongoing pregnancy rate after the antagonist is administered [27]. However, another study reported different results [28]: at the periovulatory phase, a fixed GnRH antagonist regimen showed that a low LHOTD was associated with low ongoing pregnancy and high miscarriage rates [29]. This was in contrast to the research conducted by Ramachandran et al. [30], who reported that the LHOTD was not related to pregnancy outcomes. With regard to LH levels during the process of ovarian stimulation, there was a study in which no difference in clinical outcomes was reported between the maximal LH level during COS > 4 mIU/mL and < 4 mIU/mL groups [31], whereas another study showed that the group with a minimal LH level during COS < 0.5 mIU/mL achieved a worse prognosis [32]. Our results showed that the LHOTD was not associated with clinical outcomes in the flexible antagonist regimen group except for the group of women aged less than 35 years and the excessively suppressed LHOTD group. These findings implied that more LH may be needed in younger women or those with extremely low LH levels undergoing a flexible antagonist regimen.

Interestingly, the LHOTD showed diverse effects on outcomes in different analog regimens. The aim of both regimens was to inhibit a premature LH surge; however, the mechanisms in the regimens were different, causing the circulating LH level to be discrepant (Fig. 1A). In the agonist regimen group, the LHOTD showed a denser distribution (Fig. 1B) and a lower level compared with normal physiology. It is posited that LH is essential for follicular development [33], and its level should not be too low in the COS process [34]. Thus, the profound suppression of LH might lead to insufficient and impaired LH functions. In contrast, the LH level during COS was suppressed slightly in the flexible antagonist regimen group and showed a scattered curve (Fig. 1C). The results of LH distribution were more similar to normal physiological LH levels [35]. Adequate LH levels thus support the entire COS and subsequent reproductive process, which might explain why the LHOTD with the antagonist regimen showed no correlation with the clinical pregnancy and live birth outcomes.

The agonist regimen inhibited ~ 90% of patient LH levels to a limit of < 1.34 mIU/mL, while 90% had an LH level > 1 mIU/mL in the antagonist regimen (Fig. 2). These results might be caused by the diverse mechanisms of pituitary suppression using agonists or antagonists. A GnRH agonist has an extremely high affinity for the GnRH receptor relative to wild-type GnRH in the pituitary gland, and after the transient surge of gonadotropin, the pituitary gland becomes desensitized to GnRH and stops secreting endogenous LH for a long period [36]. However, this process is not easy to control, as the pituitary response to agonists is specific to the individual. Thus, a patient’s pituitary gland may be profoundly suppressed by an agonist causing low LH levels, and these patients may be more suitable for exogenous LH supplementation. In contrast, GnRH antagonists can reversibly bind to the GnRH receptor [37] and inhibit the signaling pathway regulating gonadotropin secretion. The suppression process is mild, controlled, and transient, and the occurrence of LH deficiency is therefore less likely. Further experiments will need to clarify the reasons for these findings.

## Conclusions

In summary, the LHOTD showed a different distribution between agonist and antagonist regimens. LH was profoundly suppressed in the agonist regimen, and its level was positively correlated with the clinical pregnancy and live birth rates. In contrast, in the flexible antagonist regimen, the LHOTD was significantly higher than that in the agonist regimen and did not correlate with the outcomes, except for those of women in the nonadvanced age group and women with an excessively suppressed LHOTD. Further investigation is required to determine the rationale for these findings.

## Data Availability

The original contributions presented in the study are included in the article material. Further inquiries can be directed to the corresponding authors.

## Abbreviations

LHOTD: Luteinizing hormone level on ovulation trigger day
IVF: In vitro fertilization
GnRH: Gonadotropin-releasing hormone
ET: Embryo transfer
T1: 1St tertile
T2: 2Nd tertile
T3: 3Rd tertile
OR: Odds ratio
CI: Confidence interval
SD: Standard deviation
LH: Luteinizing hormone
FSH: Follicle-stimulating hormone
COS: Controlled ovarian stimulation
LHCGR: Luteinizing hormone/chorion gonadotrophin receptor
PCOS: Polycystic ovary syndrome
AMH: Anti-Müllerian hormone
E2: Estradiol
EMT: Endometrial thickness
hCG: Human chorionic gonadotropin
BMI: Body mass index
AFC: Antral follicle counts
